# Rad7 E3 Ubiquitin Ligase Attenuates Polyubiquitylation of Rpn10 and Dsk2 Following DNA Damage in *Saccharomyces cerevisiae*

**DOI:** 10.4236/abc.2015.57021

**Published:** 2015-12-16

**Authors:** Joseph M. Benoun, Danielle Lalimar-Cortez, Analila Valencia, Adriana Granda, Destaye M. Moore, Eric P. Kelson, Paula L. Fischhaber

**Affiliations:** Department of Chemistry and Biochemistry, California State University Northridge, Northridge, CA, USA

**Keywords:** Ubiquitin, Rad7, Rpn10, Rpn11, Dsk2, DNA Damage Response

## Abstract

During Nucleotide Excision Repair (NER) in the yeast *S. cerevisiae*, ubiquitylation of Rad4 is carried out by the E3 ubiquitin ligase that includes Rad7-Elc1-Cul3 and is critical to optimal NER. Rad7 E3 activity targets Rad4 for degradation by the proteaseome but, in principle, could also trigger other DNA damage responses. We observed increased nuclear ubiquitin foci (Ub-RFP) formation in *S. cerevisiae* containing a Rad7 E3 ligase mutant (*rad7SOCS*) in response to DNA damage by benzo[a]pyrenediolepoxide (BPDE) in dividing cells. Immunoblots reveal that ubiquitin conjugates of Rpn10 and Dsk2 accumulate in greater abundance in *rad7SOCS* compared to *RAD7* in dividing cells in response to BPDE which makes Rpn10 and Dsk2 candidates for being the ubiquitylated species observed in our microscopy experiments. Microscopy analysis with strains containing Dsk2-GFP shows that Dsk2-GFP and Dsk2-GFP/Ub-RFP colocalized in nuclear foci form to an increased extent in a *rad7SOCS* mutant background in dividing cells than in a *RAD7* wild-type strain. Further, Dsk2-GFP in the *rad7SOCS* strain formed more foci at the plasma membrane following BPDE treatment in dividing cells relative to strains containing *RAD7* or a *rad7Δ* deletion mutant. In response to a different agent, UV irradiation, levels of ubiquitylated proteins were increased in *rad7SOCS* relative to *RAD7*, and the proteasomal deubiquitylase subunit, Rpn11 was even monoubiquitylated in the absence of damaging agents. Together these data show that Rad7 E3 activity attenuates ubiquitylation of proteins regulating the shuttling of polyubiquitylated proteins to the proteasome (Dsk2 and Rpn10) and removal of ubiquitin chains just prior to degradation (Rpn11). Since Rad7 E3 ligase activity has been shown to increase ubiquitylation of its target proteins, yet our results show increased ubiquitylation in the absence of Rad7 E3, we suggest that Rad7 E3 action regulates ubiquitin ligase and deubiquitylase (DUB) activities that act on Rpn10, Dsk2 and Rpn11.

## 1. Introduction

Nucleotide Excision Repair (NER) in eukaryotes repairs DNA damage via a multistep pathway functioning in two modes with differing DNA damage recognition steps: global genome repair (GGR) and transcription-coupled repair (TCR). In GGR in *S. cerevisiae*, the DNA lesion is recognized by the Rad4-Rad23 complex. Damage recognition is followed by unwinding of the DNA surrounding the damage and damage verification by multiprotein complex TFIIH, followed by assembly of the NER complex by Rad14 and RPA. Complex assembly is followed by 5′ and 3′ incisions of the damaged strand by the Rad1-Rad10 complex and Rad2 which is interweaved with gap-filling by DNA polymerase [1] [2]. The NER pathway is largely conserved in eukaryotes, making *S. cerevisiae* a valuable model system for the regulation of NER activity.

Rad4 is ubiquitylated in response to DNA damage by the E3 ubiquitin ligase complex that includes Rad7 (Rad7-Elc1-Cul3) targeting Rad4 for degradation by the proteasome [3]. It is currently unknown if ubiquitylation of Rad4 may serve other roles. In humans, for example, the Rad4 ortholog, XPC becomes ubiquitylated during repair, but ubiquitylation increases DNA binding activity, especially on UV-damaged DNA, rather than targeting XPC for degradation [4]. Further, the Rad7-Rad16 complex along with ABF1 plays roles in histone modification and creates the torsional strain in DNA required for NER [5]-[7]. Prior reports have shown that the Rad23 ubiquitin like domain (UBL) binds the proteasome and influences NER, by mechanisms that are not yet clear [3] [8]. Numerous proteins are ubiquitylated in response to DNA damage, including several proteasome receptors and shuttle factors including Rpn10, Dsk2 and Rad23, which become ubiquitylated in order to regulate the recognition and shuttling of polyubiquitylated proteins destined for degradation by the proteasome [9]–[11].

Dsk2, Rad23, and Ddi1 are three UBL/UBA-containing shuttle factors responsible for recruiting K48-linked ubiquitylated proteins to the proteasome for destruction [12] [13]. Dsk2 and Rad23 also function in cell cycle regulation, enabling spindle pole body (SPB) duplication at the G1/S boundary and working with the Anaphase Promoting Complex to enable progression through anaphase [14] [15]. *S. cerevisiae* cells deleted of *DSK2, RAD23* and *DDI1* exhibit a delay in cell cycle progression in M phase indicating an essential role in cell cycle regulation and some genetic redundancy in their functions [15]. A *dsk2Δ rad23Δ* double mutant exhibits a G2/M arrest phenotype while a *dsk2Δ rad23Δ ddi1Δ* triple mutant additionally exhibits duplicated SPB’s that have not separated [15] [16]. Hence, Dsk2, Rad23 and Ddi1 may promote progression through M phase by aiding removal of SPB bridge proteins so that spindle poles can separate. Importantly, Dsk2 and Rad23 are ubiquitylated in response to DNA damage [17] [18]. Ubiquitylation of Rpn10 alters its binding to other polyubiquitylated proteins being recruited to the proteaseome for degradation [8] [10]. Monoubiquitylation of Rpn10 promotes its dissociation from the proteasome, which, in turn, facilitates binding of Dsk2 [19]. Interestingly *rad23Δ rpn10Δ* double mutants exhibit sensitivity to cellular stress, cell cycle delays and defective SPB duplication while Dsk2 overexpression blocks SPB duplication [20] [21]. Overexpression of either Rad23 or Rpn10 will rescue sensitivity to cellular stress, but not in the case of a Rad23 mutant that lacks two phosphorylation sites in the UBL domain [22]. Therefore, Rad23, Dsk2 and Rpn10 play key roles in cell cycle progression, which is regulated in part by their own states of ubiquitylation.

Roles for NER factors in regulating proteolytic activity and cell-cycle regulation have also been shown. Several studies showed that the proteasome negatively regulates NER through mechanisms involving Rad4 and Rad23 [3] [23]-[26]. In a screen for mutants giving rise to yeast spindle morphogenesis phenotypes, *bim1Δ rad4Δ* and *bim1Δ rad7Δ* double mutants exhibited increased delayed anaphase over *bim1Δ* single mutants [27]. The *bim1Δ rad7Δ* double mutant also exhibited delayed nuclear positioning and misoriented spindles, while a *rad4Δ* single mutant exhibited delayed nuclear positioning [27]. Bim1 is a component of the cortical microtubule capture site that physically interacts with the APC and is required for nuclear migration and the assembly and disassembly of spindle poles [28] [29]. These results indicate NER factors Rad4 and Rad7 play an ill-defined role in spindle checkpoint regulation. Additionally, mutants of the proteasome regulatory particle deubiquitylase (DUB), Rpn11 exhibit UV-sensitive phenotypes and cell cycle-arrest in M phase, suggesting that Rpn11 plays multiple roles in the UV damage response. Mutants in the metalloprotease motif of Rpn11 have been shown to exhibit not only UV sensitivity, but also defects in tubular organization and cell cycle-arrest [30]. Recent reports show that Rpn11 and Ubp6, another DUB, are situated near each other in the proteasome lid and work together to trim ubiquitin chains prior to proteasomal degradation [31] [32].

Given prior reports showing *RAD7* involvement in cell cycle and its known role as the E3 ubiquitin ligase ubiquitylating Rad4 in NER, it is of interest to determine whether Rad7 might exert a DNA damage response that regulates the proteasome in a manner that might reinforce cell cycle checkpoints following DNA damage. The present study demonstrates that a *RAD7* mutant lacking only the E3 ligase function exhibits several phenotypes consistent with a role in regulation of the ubiquitylation states of Dsk2, Rpn10 and Rpn11. These phenotypes were observed in the context of damaging agents giving rise to DNA damage only repaired by NER, strongly suggesting an NER-dependent DNA damage response. Connections between NER and checkpoint activation have been reported in the literature, but thus far indicate primarily a role for NER in recruitment of checkpoint activation kinases and the 9-1-1 clamp to DNA damage sites [33]. Our observations suggest that *RAD7* plays other roles that have not been previously characterized.

## 2. Materials and Methods

### 2.1. Preparation of Yeast Strains *Ub-RFP*, and *rad4Δ*

The *S. cerevisiae UB14* gene was tagged at the N-terminus with the gene for monomeric red fluorescent protein (mRFP) from *Discoma* [34] to prepare a strain containing fluorescently-labeled ubiquitin (Ub-RFP) in the W303-1A genetic background by adaptamer-mediated PCR [35]. All strains used in this study are detailed in Table 1. The resulting strain was crossed to produce strains PF038-1D and PF040-3A used in microscopy experiments. The presence of the RFP tag in frame with no mutations was confirmed by PCR, fluorescence microscopy and sequencing. Functional Ub-RFP was confirmed by immunoblotting of yeast WCEs using standard methods with a ubiquitin antibody (Ubiquitin (P4D1) Mouse mAb, Cat No 3936, Cell Signalling, Danvers, MA) and *α*-*β*-actin (Abcam ab8224, Cambridge, MA).

A yeast strain containing a Rad7 E3 ligase mutant (*rad7SOCS*) was prepared by cloning and site-directed mutatgenesis of *RAD7* by mutating amino acids L168 and C172 to alanines. Plasmids were constructed via adaptamer-mediated PCR and mutagenized using the Quickchange II XL kit (Agilent) and manufacturer’s instructions. Yeast transformation and backselection were carried out as referenced above. Successful backselected clones were sequenced confirming the presence of the *rad7SOCS* mutations and no additional mutations. The resulting strain was crossed to prepare strains PF084-7A and PF090-1D.

A strain containing a *RAD4* disruption was prepared by standard targeted integration of the *rad4::URA3* cassette from plasmid pNF416 [36]. A *rad4::URA3*^+^ transformant was crossed to produce strain PF097-2C. UV survival experiments confirmed identical UV sensitivity to previously published *rad4Δ* strains.

### 2.2. Preparation of Diploid Strains Containing *Dsk*2*-GFP Ub-RFP* and *Rpn*10*-GFP Ub-RFP*

Diploid strains were prepared by mating strains YMR276W and YHR200W each with PF039-2D, PF087-2C and PF125-23A to yield a panel of diploid yeast strains containing *Dsk2-GFP Ub-RFP RAD7* (PF165), *Dsk2-GFP Ub-RFP rad7SOCS* (PF166), *Dsk2-GFP Ub-RFP rad7Δ* (PF167), *Rpn10-GFP Ub-RFP RAD7* (PF162), *Rpn10-GFP Ub-RFP rad7SOCS* (PF163), and *Rpn10-GFP Ub-RFP rad7Δ* (PF164) Diploids were selected on SC agar lacking methionine and lysine and diploid status confirmed by the presence of the GFP and RFP chromophores.

### 2.3. General Microscopy

Microscopy was conducted on a Zeiss AxioImager M1 microscope with a Plan—Apochromat 100×, 1.4 numerical aperture (NA) objective oil immersion lens as previously reported [37]. Except for timelapse experiments, Ub-RFP exposure times were 800 ms. Unless indicated, images were acquired as Z-stacks of 11 images with a focal plane offset of 0.3 μm per slice. Foci were counted by manually inspecting images for regions of bright, punctate fluorescence. Images were contrast-enhanced to make the fluorescent foci visible. Data represent numbers of foci observed per cell from at least 100 cells per condition unless otherwise stated.

### 2.4. Induction of Foci with N-acetoxy-2-acetylaminofluorene (AAAF) or Benzo[a]pyrene-r-7, t-8-dihydrodiol-t-9,10-epoxide(±), (anti) (BPDE)

Cultures for AAAF or BPDE pulse-chase experiments were propagated in SC medium supplemented with 200 μg/mL Adenine (SC + ade) at 23 °C. Overnight cultures were freshly diluted, incubated (3 h) induced with AAAF or BPDE (10 μM final) or mock induced with ethanol (AAAF control) or tetrahydrofuran (BPDE control). Aliquots of cultures were processed for microscopy as previously described [37].

### 2.5. Timelapse Pulse-Chase Experiments

To manage photobleaching in timelapse experiments, cells were imaged as Z-stacks containing only three slices (as opposed to 11 as in other experiments). Foci were counted in the second slice, using the first and third to verify the presence of the focus in the second. Accordingly, foci counts for these experiments represent only about 10% of each cell thickness. YFP images were acquired using 400 ms for the 0 – 30 min timepoints and 800 ms for the 35 and 40 min time points. RFP exposure times were 200 ms for 0 – 30 min, then 400 ms for 35 and 40 min time points. 600 cells per condition were analyzed.

### 2.6. DAPI Stained Images

4′,6-diamidino-2-phenylindole (DAPI) stained images were prepared as previously reported [37].

### 2.7. Immunoblots of Cell Cycle-Arrested WCEs Treated with BPDE

Log phase cultures of strains PF038-1D, PF084-7A, PF097-2C and MGSC104 were synchronized by incubation with nocodazole, (10 μg/mL, 1 hr, 23 °C), released back into cell cycle by filtering, washing and incubation in fresh SC + ade medium (5 min, 23 °C). Released cells were DNA damage-induced (or not) by addition of BPDE to 10 μM and swirling (3 min). Cells were filtered, washed, resuspended in freshly prepared native lysis buffer (25 mM Tris-HCl, pH 8.0, 250 mM NaCl, 5 mM Na_2_EDTA, 10% Glycerol, 1 μM Phenylmethylsulfonyl fluoride, 2.5 μM Benzamidine, 0.7 μM Leupeptin, 1.45 μM Pepstatin, 5 μM *β*-Mercaptoethanol) and bead-beaten to prepare WCEs, which were quickly centrifuged to remove beads and cellular debris and snap-frozen. Aliquots normalized for total protein content were analyzed by standard SDS-PAGE and immunoblotting. Primary antibodies were obtained commercially [AbCam (Rpn10, Dsk2, PCNA [PC10], Rpn1, Rpt1, RNAPII-CTD, H2B, H3K79), Active Motif (H2A), Cell Signaling (ubiquitin), Santa Cruz (*β*-actin)] or were gifts of the laboratory of Errol Friedberg (Rad4, Rad23, Rad7 and Rad16).

### 2.8. Immunoblots of Cell Cycle-Arrested WCEs Prepared under Denaturing Conditions (for Ni-NTA Affinity Capture Experiments)

Cells transformed with plasmids YEplac195 or YEplac195 CUP1::His7-Ub were cultured in Synthetic Complete medium lacking uracil and supplemented with 200 mg/L adenine (SC-ura+ade) + 10 μM CuSO_4_ (12 h), cell cycle-arrested with nocodazole, released briefly back into cell cycle by filtering, washing and incubation in fresh SC-ura + ade medium (5 min, 30 °C) and DNA damage-induced with BPDE as described above. Cells were centrifuged, resuspended in denaturing lysis buffer (20% TCA w/v), bead-beaten, centrifuged and protein pellets resuspended in urea binding buffer (20 mM sodium phosphate, 500 mM NaCl, 8 M urea, pH 7.8), vortexed and filtered. WCEs were subjected to standard affinity capture under denaturing conditions on Ni-NTA agarose (Invitrogen) using manufacturer’s instructions. Eluates were analyzed by SDS-PAGE and immunoblotting. Primary antibodies were obtained commercially [AbCam (Rpn10, Dsk2) and Genscript (*α*-TAP)].

### 2.9. Immunoblots of Cell Cycle-Arrested, UV-Treated WCEs Prepared under Nondenaturing Conditions

Strains PF038-1D, PF084-7A and MGSC104 were cultured in YPD, cell cycle-arrested with nocodazole, released briefly back into cell cycle by filtering, washing and incubation in fresh YPD (5 min, 30 °C), centrifuged to remove medium, resuspended in a small volume of water (15 mL), transferred to a sterile petri dish and DNA damaged with UV-C (100 J/m^2^). Damaged cells were centrifuged and resuspended in native lysis buffer prior to cell disruption and processing as described above in section 2.7. Aliquots normalized for total protein content were analyzed by SDS-PAGE and immunoblotting with an ubiquitin primary antibody (Cell Signaling).

### 2.10. Immunoblots of Cell Cycle-Arrested WCEs Prepared under Nondenaturing Conditions

Strains YFR004W and PF168 were transformed with plasmids YEplac195 or YEplac195 CUP1::His7-Ub, cultured in Synthetic Complete medium lacking uracil and supplemented with 200 mg/L adenine (SC-ura + ade) + 10 μM CuSO_4_ (12 h), cell cycle-arrested with nocodazole, released briefly back into cell cycle by filtering, washing and incubation in fresh SC-ura + ade medium (5 min, 30 °C), centrifuged to remove medium, resuspended in denaturing lysis buffer (20% TCA w/v), bead-beaten, centrifuged and protein pellets resuspended in urea binding buffer (20 mM sodium phosphate, 500 mM NaCl, 8 M urea, pH 7.8), vortexed and filtered. WCEs were subjected to affinity capture under denaturing conditions on Ni-NTA agarose (Invitrogen) using manufacturer’s instructions. Eluates were analyzed by SDS-PAGE and immunoblotting with an antibody to TAP (Genscript).

## 3. Results

### 3.1. Ubiquitin Foci Form in the Nucleus in Response to AAAF and BPDE Treatment

**Figure 1:**
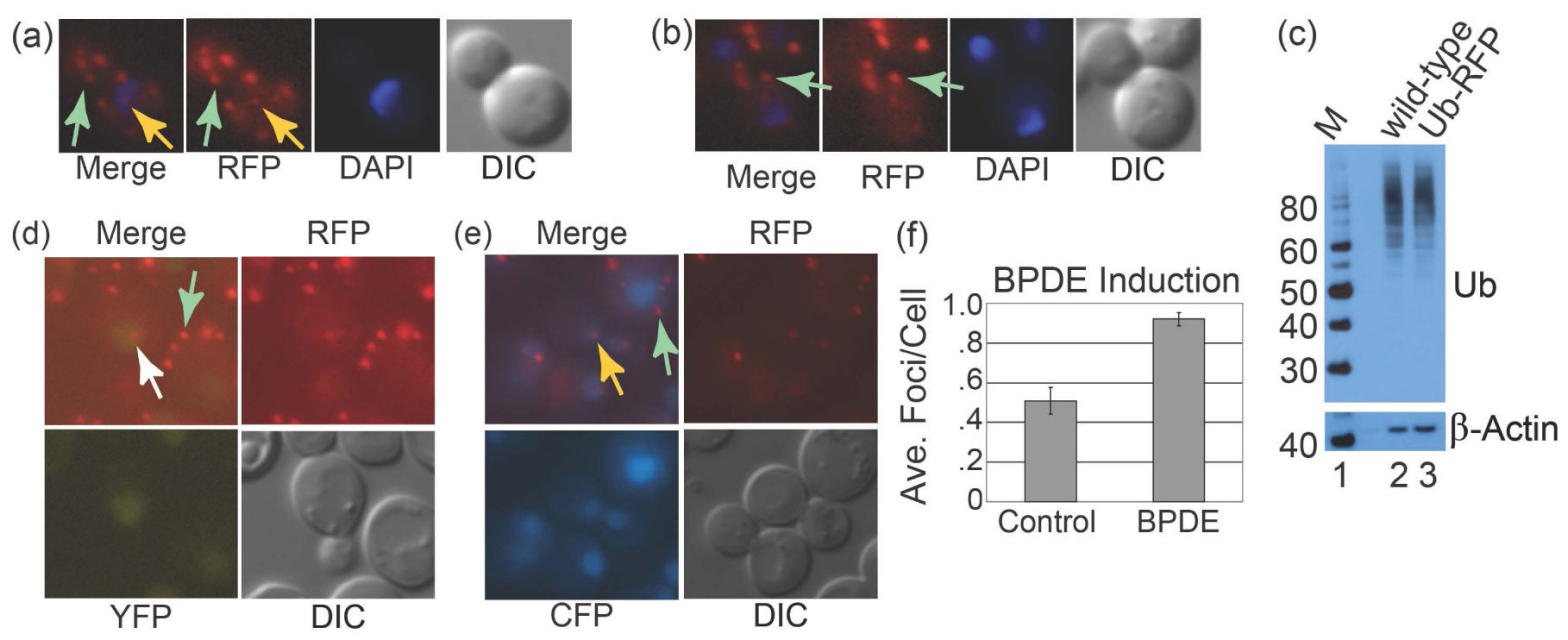
(a) Merged RFP and DAPI fluorescent images are shown (left) of a Ub-RFP cell 15 min following induction with UV-C (20 J/m^2^) followed by images containing only RFP and DAPI channels (middle) and a differential interference contrast (DIC) image (right). Bright cytosolic RFP foci are visible throughout the cell (green arrow indicates one focus). A nuclear-localized RFP focus is indicated (orange arrow); (b) Same as (a) except cell shown does not contain a nuclear-localized RFP focus. Bright cytosolic RFP foci are visible throughout the cell (green arrow indicates one focus); (c) Immunoblot of WCEs from the indicated yeast strains probed with an antibody to ubiquitin (Ub). Lower panel shows the same blot reprobed with an antibody to β-Actin. Lane 1: MagicMark^™^ protein standard, lane 2: wild-type (W1588-4C), lane 3 *Ub-RFP* PF038-1D); (d) The *Ub-RFP Rad10-YFP* strain (PF038-1D) was incubated with BPDE (10 μM, 1 h) and imaged as a single focal plane. Merged, RFP, YFP and DIC images are shown. A white arrow indicates a nuclear-localized RFP focus; (e) Same as panel (c), except the strain was *Ub-RFP Rad14-CFP* (PF040-3A) and the DNA damaging agent was AAAF. A nuclear-localized RFP focus is indicated with an orange arrow. In both (d) and (e), green arrows indicate examples of RFP foci that are not nuclear or colocalized; (f) Graph of nuclear Ub-RFP foci counts from 11-slice Z-stacks (rather than single focal plane images) acquired during experiments described/depicted in panel (d). Data are shown for *Ub-RFP Rad10-YFP* strain (PF038-1D) samples induced with BPDE (10 μM, 1 h) or controls mock-induced with tetrahydrofuran (THF). Error bars represent standard error.

To investigate the role of Rad7 in the DNA damage response, the *Saccharomyces cerevisiae* ubiquitin gene (*UB14*) was N-terminally-tagged with monomeric red fluorescent protein (Ub-RFP). Following treatment of Ub-RFP cells with Benzo[a]pyrenediolepoxide (BPDE) or N-Acetoxy-2-acetylaminofluorene (AAAF), RFP foci were formed in the nucleus that colocalized with the fluorescent signal from DAPI (Figure 1(a), orange arrows). This strain also exhibits large cytosolic RFP foci with or without DNA damage and their brightness and numbers did not appear to change significantly following DNA damage, but this was not investigated rigorously by us (Figure 1(a), and Figure 1(b) green arrows). However, a prior RFP screen indicated that an analogous Ub-RFP strain exhibited both pan-nuclear and pancytosolic localization of Ub-RFP, which contrasts with our findings [38]. Based on the classification system used in the prior report, our cytosolic foci could correspond to ubiquitin localized any of a number of cytosolic features including Golgi, peroxisomes, endosomes or actin but this was not examined by us. To confirm the functionality of the Ub-RFP strain we analyzed WCEs for high molecular weight (HMW) ubiquitylated proteins and found similar band intensities when comparing wild-type *UB14* and *Ub-RFP* strain extracts (Figure 1(c), upper panel). A β*-*actin loading control shows similar loading between *UB14* and *Ub-RFP* (Figure 1(c), lower panel).

We investigated DNA damage responses using strains containing *Ub-RFP* and either the *Rad10-YFP* or *Rad14-CFP*. Strains bearing these fluorescently labeled NER genes exhibit pan-nuclear YFP or CFP signal in the absence of DNA damage allowing identification of the location of nucleus within the cell [37] [39]. Following DNA damage, YFP and CFP signal is still pan-nuclear but bright, punctate YFP or CFP foci are induced within the nucleus following a temporal delay, thereby enabling the YFP and CFP signal to reveal the location of the nucleus within the cell but also indicating sites of NER within the nucleus [37] [39]. The *Ub-RFP Rad10-YFP* strain was treated with BPDE and imaged, exhibiting Ub-RFP foci localized within the periphery of the pan-nuclear Rad10-YFP signal (Figure 1(d)). Analogous experiments with the *Ub-RFP Rad14-CFP* strain and AAAF as the DNA damaging agent similarly showed Ub-RFP foci localized within the nuclear periphery (Figure 1(e)). Images of both damaged and undamaged cells showed the aforementioned, large, bright cytosolic Ub-RFP foci, which, as stated, did not change appreciably following BPDE and AAAF treatment (Figure 1(d) and Figure 1(e), green arrows). Images from *Ub-RFP Rad10-YFP* BPDE experiments were analyzed by counting the nuclear-localized Ub-RFP foci and which exhibited approximately 1.8-fold induction (Figure 1(f)). Attempts at Ub-RFP induction in response to UV were not successful owing to bleaching of the Rad10-YFP and Rad14-CFP fluorophores (data not shown). Together these data show that nuclear ubiquitylated species form in response to BPDE and AAAF, two agents giving rise to damage only repaired by NER.

### 3.2. Nuclear Ubiquitin Foci Form Rapidly in Response to BPDE Treatment in a *RAD7* E3 Ubiquitin Ligase Mutant

**Figure 2:**
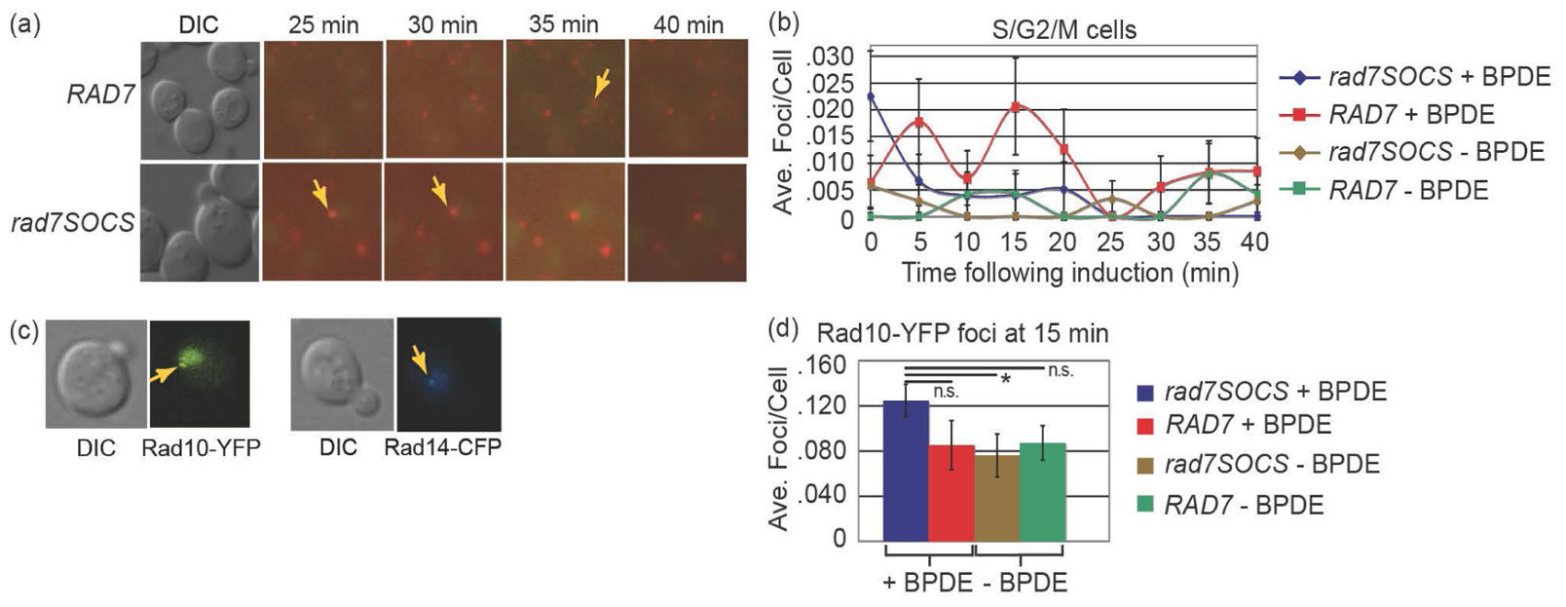
Timelapse experiments with the *Ub-RFP Rad10-YFP* strains following BPDE treatment and imaging at 5 minute intervals. Representative DIC and merged RFP/YFP images are shown from strains containing either the *RAD7* (PF038-1D, upper images) or *rad7SOCS* (PF084-7A, lower images). Gold arrows indicate nuclear-localized Ub-RFP foci. Note in the lower panels a Ub-RFP focus is shown which persisted through two timepoints. (b) Quantification of timelapse experiments. (c) Representative images from *RAD10-YFP* and *RAD14-CFP* strains showing pan-nuclear YFP or CFP signal and a Rad10-YFP or Rad14-CFP focus. Gold arrows indicate nuclear-localized foci. (d) Quantification of Rad10-YFP foci observed at 15 min in timelapse experiments. In (b) and (d), the plotted values represent means (*λ*’s) from ideal Poisson distributions least squares fit to the observed distributions of foci per cell. The least squares deviations were propagated into the overall errors, which are depicted as the error bars in the graphs.

The *Ub-RFP Rad10-YFP* strain was monitored as a function of time to determine the timescale for maximum induction and duration of focus persistence. Nuclear Ub-RFP foci were observed appearing and disappearing in successive images of the same fields of cells during 5 minute intervals acquired immediately following BPDE treatment (Figure 2(a), upper panels). To examine whether the nuclear Ub-RFP foci observed were NER foci containing ubiquitylated Rad4, we prepared a *RAD7* mutant containing two amino acid substitutions abrogating the Rad7 E3 ubiquitin ligase activity that would normally ubiquitylate Rad4 (*rad7SOCS*) [3]. BPDE induction experiments with *rad7SOCS* similarly exhibited nuclear localized Ub-RFP foci (Figure 2(a), lower panels). When foci counts of S, G2 and M cells from *RAD7* and *rad7SOCS* strains were compared, nuclear Ub-RFP foci were in greater abundance at the earliest time point in *rad7SOCS* (Figure 2(b)). We observed approximately 0.023 nuclear foci per cell in *rad7SOCS* at zero min with the signal becoming more diminished in successive time points while *RAD7* cells exhibited 0.005 foci at zero minutes and peaked at approximately 0.020 nuclear foci per cell at 15 min post-induction (Figure 2(b)). The “zero minutes” timepoint reflects when cells were harvested following BPDE treatment, but processing for microscopy takes approximately three minutes. The Ub-RFP foci observed in these experiments are extremely rare events, even at peak focus induction, in part because only a fraction (~1/3) of each cell is being imaged. Hence, to ensure meaningful signal-to-noise, we analyzed a minimum of 600 cells for each condition at each time point.

Our observation of increased abundance of Ub-RFP foci in *rad7SOCS* relative to *RAD7* at zero minutes suggested that the foci were not NER foci since, in principle, the inability to ubiquitylate Rad4 would ostensibly result in *fewer* ubiquitylated NER protein complexes in *rad7SOCS*, relative to *RAD7*. To further cull the data for possible NER foci, YFP and CFP images acquired in parallel with Ub-RFP following BPDE and AAAF damage were inspected for colocalized Ub-RFP/Rad10-YFP and Ub-RFP/Rad14-CFP foci and none were observed (data not shown). For reference, examples of typical Rad10-YFP and Rad14-CFP foci are shown (Figure 2(c), gold arrows). Since DNA damaging agents have been shown to induce Rad10-YFP foci following a temporal delay [37] [39], as a control, we also quantitatively analyzed images from Rad10-YFP/Ub-RFP/BPDE experiments at 15 minutes for Rad10-YFP foci. Numbers of Rad10-YFP foci were not dramatically altered in response to BPDE treatment, with the BPDE-treated *rad7SOCS* cells exhibiting a slight induction (~1.5-fold) of Rad10-YFP foci relative to uninduced controls while no induction was observed in *RAD7* (Figure 2(d)). In summary, only limited Rad10-YFP focus induction was observed after 15 minutes and the temporal pattern differed from that of Ub-RFP.

### 3.3. Polyubiquitylated Rpn10 and Dsk2 Are Present at Increased Levels Following BPDE Treatment in a *RAD7* E3 Ubiquitin Ligase Mutant

Both the pattern of Ub-RFP focus induction and the lack of induction of Ub-RFP/Rad14-CFP or Ub-RFP/ Rad10-YFP colocalized foci suggested the Ub-RFP foci were not NER foci, so we performed experiments to determine the molecular basis for the nuclear ubiquitin-RFP foci. We hypothesized that nuclear Ub-RFP foci may derive from ubiquitylated histones, PCNA, or components of the proteasome [10] [40]–[44].

**Figure 3:**
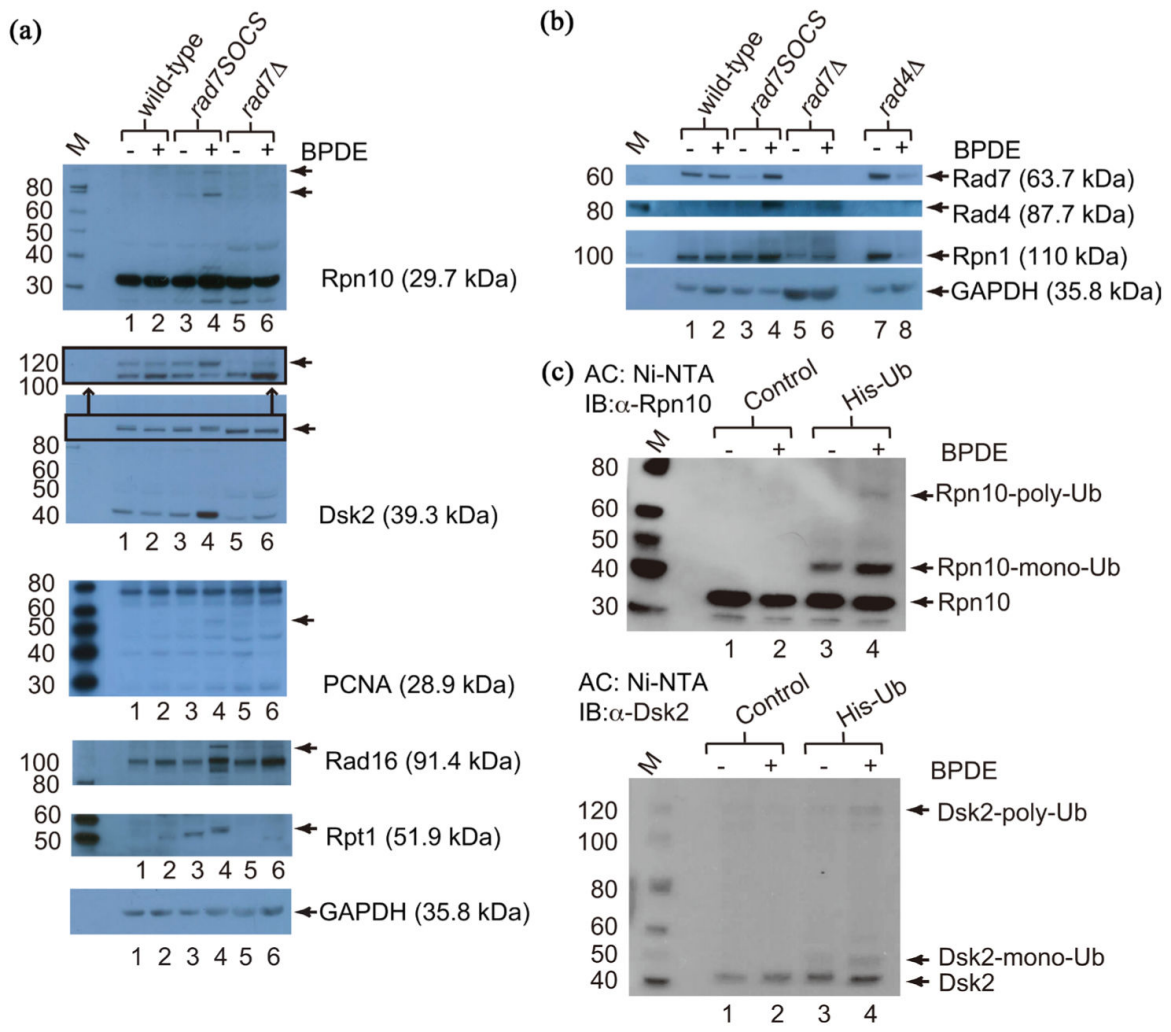
Cells from strains PF038-1D, (“wild-type”, lanes 1 and 2), PF084-7A, (“*rad7SOCS*”, lanes 3 and 4), MGSC104, (“*rad7Δ*”, lanes 5 and 6) or PF097-2C, (“*rad4Δ*”, lanes 7 and 8, panel B only) were cell cycle-arrested at the G2/M boundary, released briefly back into cell cycle, treated with 10 μM BPDE (lanes 2, 4, 6 or 8 “+”) for 3 minutes or mock treated (lanes 1, 3, 5 and 7 “-”) and disrupted. Normalized protein quantities were analyzed by SDS-PAGE and immunoblotted with the indicated antibodies. “M” indicates MagicMark^™^ molecular weight marker. The expected native protein size is shown in parentheses next to the antibody name. (a) Protein candidates for which we observed HMW species that appeared in greater abundance in *rad7SOCS*, BPDE-treated extracts as compared to controls. Arrows indicate the observed HMW species. The second image panel from the top shows a portion of a Dsk2 immunoblot from a 3% – 8% Tris-Acetate gel which gave better resolution of the corresponding HMW bands than the corresponding 4% – 20% Tris-glycine gel shown immediately below. Glyceraldehyde phosphate dehydrogenase (GAPDH) is a loading control. (b) Control immunoblots showing: (top panel) the *rad7SOCS* polypeptide in the *rad7SOCS* strain, (second panel) the accumulation of Rad4 in the absence of polyubiquitylation and degradation of Rad4 in *rad7SOCS* (compare lane 4 with lanes 1 – 3), (third panel) Rpn1 and (bottom panel) GAPDH as a loading control. (c) Ni-NTA affinity capture experiments from cells transformed either with the empty vector (YEplac 195, “Control”, lanes 1 and 2) or with YEplac195 CUP1::His7-Ub (“His-Ub”, lanes 3 and 4), cell cycle-arrested, BPDE-treated and harvested under denaturing conditions prior to Ni-NTA affinity capture. Immunoblots for Rpn10 (top panel), or Dsk2 (bottom panel) are shown.

To test these hypotheses we prepared yeast WCEs from isogenic *RAD7* wild-type and *rad7SOCS* strains cell cycle-arrested at G2/M, released briefly into cell cycle and DNA damaged with BPDE using conditions similar to microscopy experiments. Immunoblots with antibodies to Rpn10, Dsk2, PCNA, Rad16 and Rpt1 exhibited HMW species in WCEs from *rad7SOCS* in greater abundance than *RAD7*, or uninduced controls (bands indicated with arrows in Figure 3(a)). Further, the HMW bands were not observed in a *rad7Δ* mutant strain that contained a wild-type ubiquitin gene instead of Ub-RFP (lanes 5 and 6, Figure 3(a)) indicating that their formation in higher abundance is specific to the lack of the E3 ubiquitin ligase function of Rad7 but also dependent on the presence of the of Rad7 polypeptide itself. Notably, native (unubiquitylated) Dsk2 protein abundance was also significantly increased in *rad7SOCS* following BPDE treatment (lane 4, Figure 3(a)). The observed pattern of HMW band induction aligns with results from the zero time point in our timelapse microscopy experiments (Figure 2(b)), making these HMW species candidates for being the ubiquitylated proteins in microscopy since all are nuclear-localized in dividing cells.

Membranes were immunoblotted for several other candidates including histones H2A and H2B, Rad23, Rad51, H3K79me3 and RNAPII, none of which exhibited a greater abundance of HMW bands in *rad7SOCS* following BPDE over *RAD7* or uninduced controls (Supplemental Figure S1). Notably, we observed a slight increase in H3K79me3 signal in *rad7SOCS* following BPDE treatment and a distinct signal increase in *rad7Δ* in the absence of DNA damage (Supplemental Figure S1), which could be indicative of altered checkpoint activation in *rad7SOCS* and *rad7Δ* [40]. Control representative blots were also hybridized with antibodies to Rad7, Rad4 and Rpn1, and included extracts from an isogenic *rad4Δ* strain (Figure 3(b)). Results from the Rad7 immunoblot indicate that *rad7SOCS* protein is present in lower abundance in the absence of DNA damage but, following BPDE treatment, increases to approximately the level observed in *RAD7* (Figure 3(b)). As expected, Rad4 levels are higher in the *rad7SOCS* strain following BPDE treatment since Rad4 expression is induced following DNA damage and degraded more slowly in the absence of polyubiquitylation by Rad7 (Figure 3(b)). Rpn1 levels were elevated slightly in *rad7SOCS* following BPDE and in *rad4Δ* without BPDE treatment (Figure 3(b)). Rpn1 levels were diminished in *rad7Δ* with or without BPDE treatment and highly diminished in *rad4Δ* following BPDE treatment, but we did not investigate whether the diminishment reflects degradation or loss by some other means (Figure 3(b)).

To determine whether the Rpn10 and Dsk2 HMW bands observed in BPDE-treated extracts represented ubiquitylated proteins we performed Nickel Nitriloacetic acid (Ni-NTA) affinity chromatography pull down experiments using extracts from cells transformed with a plasmid containing a His-tagged ubiquitin gene, cell cycle-arrested at the G2/M boundary, released briefly from arrest and DNA damaged with BPDE prior to cell disruption under denaturing conditions. Immunoblots with antibodies to Rpn10 and Dsk2 (Figure 3(c)) gave specific HMW bands of similar sizes as had been observed in non-His-tagged WCEs (Figure 3(a)). The stoichiometry of ubiquitin conjugates were tentatively assigned as mono- and polyubiquitylated forms based on SDS PAGE mobility. It is important to note that in experiments analyzing Ub-RFP WCE’s (Figure 3(a)), the Ub-RFP conjugates would be expected to increase the molecular weights of the native proteins in ~38 kDa increments (8 kDa Ub + 30 kDa RFP) while in Figure 3(c) the addition of the 6x His tag adds only ~8.8 kDa per His-tagged ubiquitin conjugate (8 kDa Ub + 0.84 kDa 6x His). Any polyubiquitin conjugates pulled down would be expected to have at least one 6x His-containing 8.8 kDa conjugate, but, if polyubiquitylated, could have varying mixtures of additions of 8.8 kDa plus ~38 kDa units, perhaps explaining why HMW bands in the two experiments (Figure 3(a) and Figure 3(c)) do not have exactly the same electrophoretic mobilities. Native Rpn10 and Dsk2 were pulled down in both His-tagged ubiquitin samples and untagged control experiments, possibly indicating pull down of a background of histidine-rich proteins that are constantly undergoing proteasomal degradation that bind tightly to both Ni-NTA and either Rpn10 or Dsk2 during pull down experiments, even under the denaturing conditions employed.

### 3.4. Dsk2-GFP Foci Are Formed at Increased Levels in a *RAD*7 E3 Ubiquitin Ligase Mutant

To further investigate whether ubiquitylated forms of Dsk2 and Rpn10 were nuclear localized in response to BPDE damage, we prepared yeast diploids containing *DSK*2/*Dsk2-GFP UB14*/*Ub-RFP RAD10*/*Rad10-YFP* or *RPN10/Rpn10-GFP UB14*/*Ub-RFP RAD10/Rad10-YFP* in either a *RAD7*/*RAD7*, *rad7SOCS*/*rad7SOCS* or *rad7Δ*/ *rad7Δ* strain background and carried out BPDE damage induction experiments on cells synchronized at the G2/M boundary and release briefly back into cell cycle.

**Figure 4:**
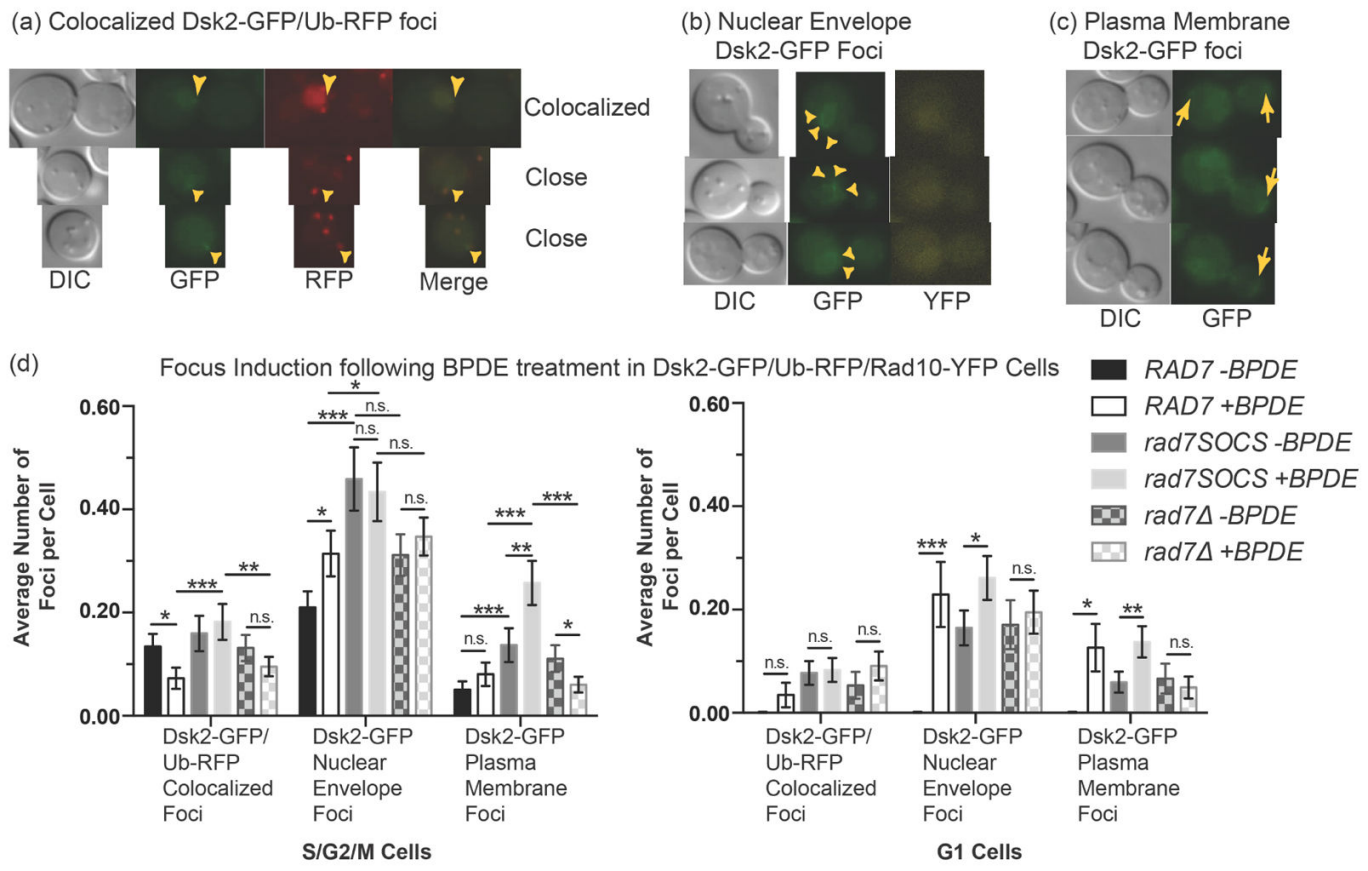
Fluorescence microscopy in Dsk2-GFP/Ub-RFP/Rad10-YFP diploid strains [PF165 (*RAD7*), PF166 (*rad7SOCS*), PF167 (*rad7Δ*)], were cell cycle arrested and treated with BPDE. (a) Examples of nuclear colocalized and “close” Dsk2-GFP/Ub-RFP foci, which were grouped and tallied together as simply “colocalized” for the purposes of our data analysis. DIC, GFP and RFP images are shown as well as merged GFP/RFP images. (b) Examples of nuclear envelope signal in which Dsk2-GFP signal circling portions of the edge of the nuclear envelope was observed. Rad10-YFP pan-nuclear fluorescence shows the approximate location of the nucleus. (c) Examples of plasma membrane Dsk2-GFP foci. Yellow arrows indicate the locations of the foci. (d) Graph of foci counts observed in S, G2 and M phase cells in 3-slice Z-stacks acquired during experiments depicted in panels (a)-(c) (left graph) and corresponding foci counts in G1 phase (right graph). The plotted values represent means (*λ*’s) from ideal Poisson distributions least squares fit to the observed distributions of foci per cell. The least squares deviations were propagated into the overall errors, which are depicted as the error bars in the graphs. Statistical comparisons were carried out via a paired t_calc_ according to the NIST/SEMATECH e-handbook of Statistical Methods and then calculating p from integration of the single tail area beyond the paired t_calc_ in a Gaussian distribution. “***” indicates p < 0.001, “**” indicates 0.001 < p < 0.01, “*” indicates 0.01 < p < 0.05, “n.s.” indicates 0.05 < p.

In microscopy experiments with the *DSK2/Dsk2-GFP UB14/Ub-RFP RAD10/Rad10-YFP* strain panel we made several noteworthy observations. First, we observed nuclear-colocalized Dsk2-GFP/UbRFP foci (Figure 4(a)). Some Dsk2-GFP/Ub-RFP foci observed were not perfectly colocalized, but within one pixel of each other and were classified along with the others as “colocalized” (Figure 4(a)). Such foci could presumably belong to a large object such as the proteasome to which both Ub-RFP and Dsk2-GFP had localized. Second, in many cells we observed a distinct “ring” of Dsk2-GFP signal that appeared to localize to the nuclear envelope (Figure 4(b)). Third, we observed bright Dsk2-GFP foci that appeared to localize to the plasma membrane, frequently at the opposing poles that aligned with the axis of the mitotic spindle, ostensibly at the microtubule organizing center (MTOC) (Figure 4(c)). We quantified all these phenomena and found that all were observed in higher abundances in *rad7SOCS* than in either *RAD7* or *rad7Δ* in dividing cells (left graph, Figure 4(d)). In the case of Dsk2-GFP plasma membrane foci, abundances were even higher in BPDE-treated dividing cells than in uninduced controls (left graph, Figure 4(d)). G1 cells exhibited lower overall abundances of these foci, but we still observed some statistically significant differences between BPDE-treated cells over uninduced controls (right graph, Figure 4(d)). Together these data indicate that Dsk2 localization and state of ubiquitylation is influenced by Rad7 E3 activity. Further, our data suggest that *RAD7* could play more than one role in governing Dsk2 localization/ubiquitylation since different phenotypes were observed between *rad7SOCS* and *rad7Δ*. We did not observe significant differences in the appearances of images recorded for the *RPN10/Rpn10-GFP UB14/Ub-RFP RAD10/Rad10-YFP* strain panel (data not shown).

### 3.5. Ubiquitylated Proteins Form at Increased Levels in a *RAD7* E3 Ubiquitin Ligase Mutant Following UV and Monoubiquitylated Rpn11 Forms Even in the Absence of UV

**Figure 5:**
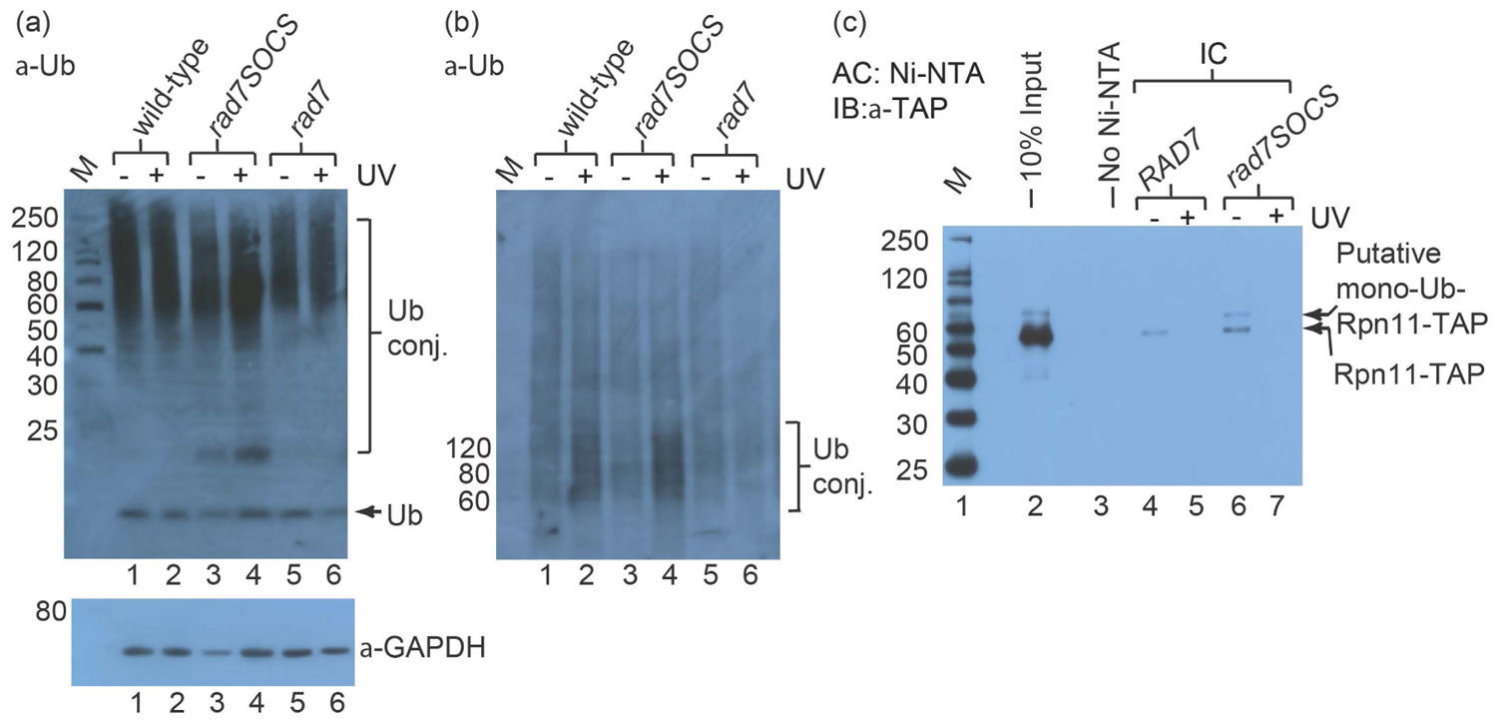
Increased levels of ubiquitylated proteins are observed in *rad7SOCS* cells following UV and increased monoubiquitylated Rpn11 is observed in *rad7SOCS* without damage. (a) Cells from strains PF038-1D, (“wild-type”, lanes 1 and 2), PF084-7A, (“*rad7SOCS*”, lanes 3 and 4) and MGSC104, (“*rad7Δ*”, lanes 5 and 6), were cell cycle arrested at the G2/M boundary, released briefly back into cell cycle, treated with 100 J/m^2^ UV-C (lanes 2, 4 or 6, “+”) for 3 minutes or mock treated (lanes 1, 3 or 5, “-”) and disrupted. Normalized quantities of protein from WCEs were analyzed on a NuPAGE Novex 4% – 12% Bis-Tris gel with MES SDS running buffer SDS-PAGE and immunoblotted with antibodies to Ub (upper panel) or GAPDH (lower panel) as a loading control. “M” indicates MagicMark^™^ molecular weight marker. (b) Same as upper panel of (a) except that extracts were analyzed on a NuPAGE Novex 3% – 8% Tris-Acetate Gel with Tris-Acetate running buffer. Arrows indicate ubiquitin; brackets indicate putative ubiquitin-conjugates. (c) The *Rpn11-TAP* strain (YFR004W) and an isogenic *Rpn11-TAP/rad7SOCS* derivative (PF168) were transformed with YEplac195 CUP1::His7-Ub, cell cycle arrested, released briefly back into cell cycle and disrupted. Denatured protein extracts were subjected to Ni-NTA affinity capture experiments. Lane 1, MagicMark^™^ molecular weight marker; lane 2, 10% input; lane 3, affinity capture in which Ni-NTA beads were omitted; lane 4, affinity capture from *Rpn11-TAP*, no UV; lane 5 affinity capture from *Rpn11-TAP* with UV; lane 6 affinity capture from *Rpn11-TAP*/*rad7SOCS*, no UV; lane 7 affinity capture from *Rpn11-TAP* with UV.

Next we examined whether ubiquitylated proteins form in greater abundance in *rad7SOCS* in response to DNA damaging agents other than BPDE. WCEs were prepared from cultures synchronized at the G2/M boundary, released briefly back into cell cycle and UV-irradiated. Immunoblots of these WCEs with the ubiquitin antibody showed increased levels of ubiquitylated species in *rad7SOCS* as compared to *RAD*7 and especially as compared to *rad7Δ*, mirroring the patterns observed in microscopy experiments and immunoblots with BPDE-treated extracts (Figure 5(a)). When HMW proteins from these extracts were resolved more fully using a 3% – 8% Tris-acetate gradient gel, it became even more clear that HMW ubiquitylated proteins were observed in greater abundance in *rad7SOCS* following UV (Figure 5(b)), compared to *rad7Δ* extracts.

Since prior reports identified the existence of UV-sensitive mutants of proteasome DUB, Rpn11 [30], we prepared an Rpn11-TAP strain panel containing either *RAD7* or *rad7SOCS*, transformed with the His-Ub plasmid and subjected protein extracts to Ni-NTA affinity capture experiments under denaturing conditions. In addition to isolating native Rpn11-TAP in both *RAD7* and *rad7SOCS* extracts, we also observed a slightly larger molecular weight band in *rad7SOCS* but not *RAD7* (Figure 5(c), lanes 4 & 6). Capture of native Rpn11-TAP, as in Figure 3(c), likely represents pull down of ubiquitylated species bound to proteasomes that had not fully denatured under the conditions in our experiment and was similar between strains. However, capture of the ~8 kDa larger Rpn11-TAP species observed in *rad7SOCS* could represent monoubiquitylated Rpn11-TAP. The presence of this band in the *rad7SOCS* but not *RAD7* extract indicates that Rad7 E3 function influences the state of ubiquitylation of Rpn11 and normally attenuates accumulation of monoubiquitylated Rpn11. We also analyzed analogous extracts that had been UV-treated (Figure 5(c), lanes 5 & 7). These UV-treated extracts were devoid of Rpn11-TAP-positive bands in both *RAD7* and *rad7SOCS* extracts but we do not know whether this reflects degradation of Rpn11 or its depletion through other means in our assay system. These data show that the Rpn11 status changed as a function of *RAD7* genetic status.

## 4. Discussion

**Figure 6:**
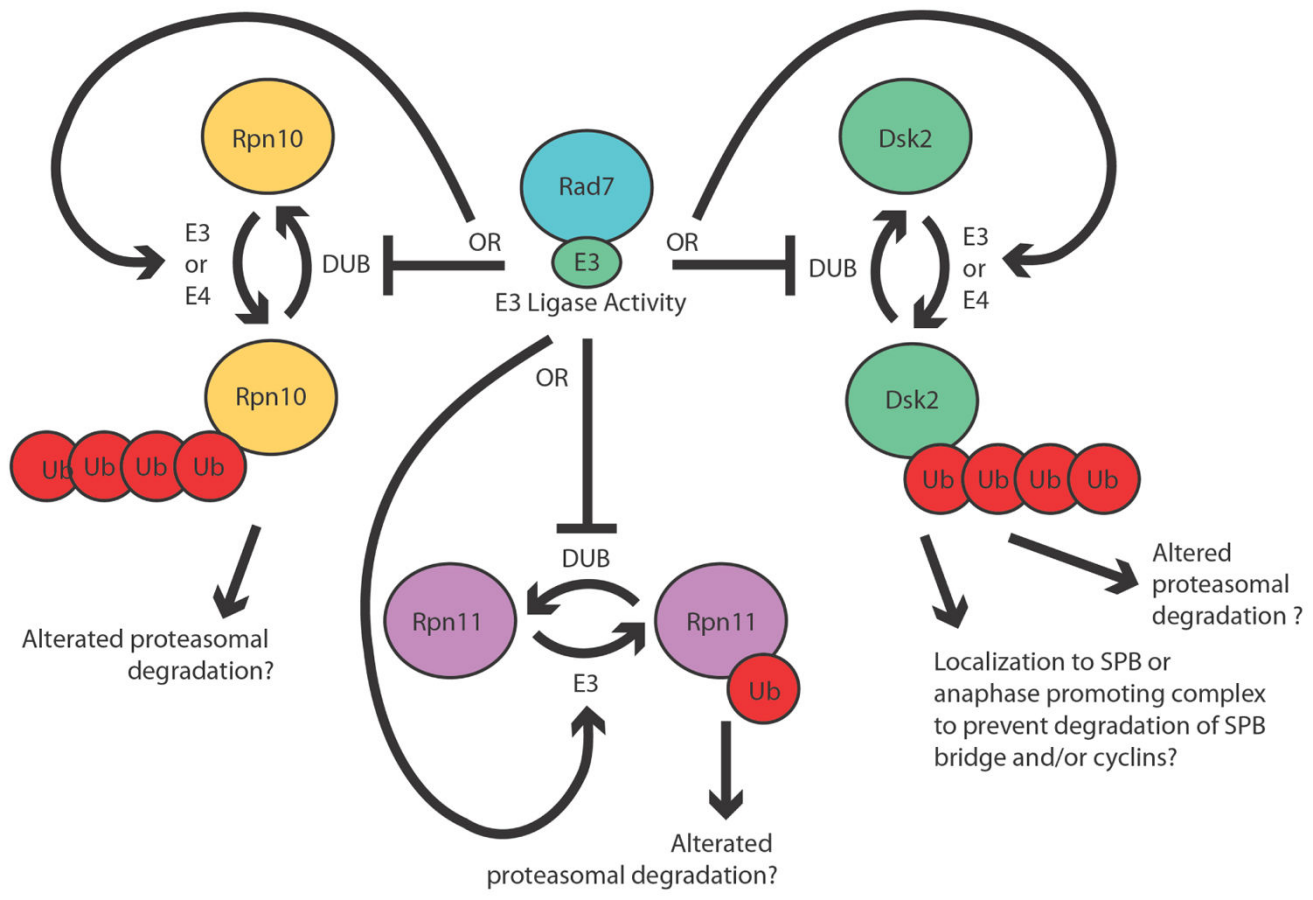
Model for regulation of proteasomal factors Rpn10, Dsk2, and Rpn11. Ubiquitylation of Rpn10, Dsk2 and Rpn11 is augmented in a *rad7SOCS* background indicating that Rad7 E3 normally attenuates ubiquitylation. We propose that Rad7 E3 ligase function may alternately either stimulate ubiquitylation or inhibit deubiquitylation of all three proteins. Such alteration may influence the amount and types of proteins degraded by the proteasome. In the case of Dsk2, alteration may also influence localization to spindle pole bodies or the APC.

Our data reveal that the E3 ligase function of *RAD7* attenuates ubiquitylation of proteasomal proteins Dsk2, Rpn10 and Rpn11 and alters localization of Dsk2 in response to DNA damaging agents. Intriguingly, we also observed changes in abundances and gel mobilities of several other proteasome regulatory particle subunits that were a function of *RAD7* or *RAD4* gene status (Rpt1 and Rpn1) providing further evidence that NER damage recognition factors interact with the proteasome following DNA damage in a complicated manner. The direct target of Rad7 E3 ligase activity responsible for all these observations remains to be identified, but our data are consistent with it being another E3 or E4 ubiquitin ligase, which is stimulated by the action of Rad7 E3, or, alternatively, a DUB which is inhibited by Rad7 E3 action (Figure 6). In principle, there could be more than one target, including a combination of E3 and E4 ligases and DUBs, and targeting could either be direct or part of a multistep cascade of which Rad7 E3 action is an upstream step.

Cell cycle checkpoints are regulated by proteasomal degradation of key proteins. There is literature precedent for participation of both Rpn10 and Dsk2 in the degradation of cyclins [10] [45]. Dsk2 and Rpn10 are genetically linked with SPB duplication and spindle pole bridge separation, two key events in cell division in *S. cerevisiae*. Dsk2 normally shuttles polyubiquitylated proteins to the proteasome, while Rpn10 provides selective access of polyubiqutylated proteins to the proteasome core for destruction. It is therefore tempting to speculate that the role of Rad7 is to regulate polyubiquitilation of Dsk2 and Rpn10, monoubiquitylation of Rpn11, and ultimately the SPB and spindle pole bridge proteins in a manner that fine tunes checkpoint activation in the face of DNA damage, perhaps depending on whether repair is proceeding effectively. In support of this possibility, we observed alterations in H3K79me3 signal in *rad7SOCS* following BPDE damage and in *rad7Δ* in the absence of BPDE (Supplemental Figure S1).

Our microscopy data support this model; *Dsk*2*-GFP Ub-RFP* diploids revealed slightly higher levels of Dsk2-GFP localized to the nuclear envelope in dividing cells (the location of proteasomes during cell division) and higher Dsk2-GFP/Ub-RFP nuclear colocalization was observed in *rad7SOCS* cells (Figure 4). We also observed an increase in abundances of plasma membrane foci in *rad7SOCS* in dividing cells following BPDE treatment, indicating a greater abundance of Dsk2 associated with a structure present at the plasma membrane during M phase and which appeared to be along the mitotic spindle axis, possibly localized to MTOCs (Figure 4). This result augurs well with the known requirement for Dsk2 in SPB separation and progression through anaphase [15] [46]. Rad7 influence on Dsk2 localization may also explain prior observations in which delayed nuclear positioning, delayed anaphase and misoriented spindles were observed in *bim1Δ rad7Δ* double mutants [27]. Taken together our results show Rad7 E3 activity influences the states of ubiquitylation of several proteasome-associated proteins.

## Supplementary Material



## Figures and Tables

**Table 1:** Strains used in this study.

Strain Name	Genotype	Where Published
MGSC104^a^	*MATα rad7Δ::LEU2 ade2-1 trp1-1 can1-100 leu2-3,112 his3-11,15 ura3-1*	[47]
PF038-1D	*MAT****a*** *ADE2 lys2Δ trp1-1 can1-100 his 3-11,15 leu2-3,112 ura3-1* *RAD10-YFP Ub-RFP*	This manuscript
PF040-3A	*MAT****a*** *ADE2 lys2Δ trp1-1 can1-100 his 3-11,15 leu2-3,112 ura3-1* *Rad14-CFP Ub-RFP*	This manuscript
PF084-7A	*MAT****a*** *ADE2 lys2Δ trp1-1 can1-100 his 3-11,15 leu2-3,112 ura3-1* *RAD10-YFP Ub-RFP rad7SOCS(L168A, C172A)*	This manuscript
PF090-1D	*MAT****a*** *ADE2 lys2Δ TRP can1-100 his 3-11,15 leu2-3,112 ura3-1 Rad14-CFP* *Ub-RFP rad7SOCS(L168A, C172A)*	This manuscript
PF097-2C	*MAT****a*** *ADE2 lys2Δ trp1-1 can1-100 his3-11,15 leu2-3,112 ura3-1* *RAD10-YFP Ub-RFP rad4::URA3*	This manuscript
PF162	*MAT****a****/MATα LYS2/lys2Δ TRP1/trp1-1 CAN1/can1-100 his 3-11,15/his3Δ1* *leu2-3,112/leu2Δ0 MET15/met15Δ0 ura3-1/ura3Δ0 RPN10/Rpn10-GFP* *RAD10/Rad10-YFP UB14/Ub-RFP*	This manuscript
PF163	*MAT****a****/MATα LYS2/lys2Δ CAN1/can1-100 his 3-11,15/his3Δ1* *leu2-3,112/leu2Δ0 MET15/met15Δ0 ura3-1/ura3Δ0 RPN10//Rpn10-GFP* *RAD10/Rad10-YFP UB14/Ub-RFP RAD7/rad7SOCS(L168A, C172A)*	This manuscript
PF164	*MAT****a****/MATα LYS2/lys2Δ CAN1/can1-100 his 3-11,15/his3Δ1* *leu2-3,112/leu2Δ0 MET15/met15Δ0 ura3-1/ura3Δ0 RPN10/Rpn10-GFP* *RAD10/Rad10-YFP UB14/Ub-RFP RAD7/rad7Δ::LEU2*	This manuscript
PF165	*MAT****a****/MATα LYS2/lys2Δ TRP1/trp1-1 CAN1/can1-100 his 3-11,15/his3Δ1* *leu2-3,112/leu2Δ0 MET15/met15Δ0 ura3-1/ura3Δ0 DSK2/Dsk2-GFP* *RAD10/Rad10-YFP UB14/Ub-RFP*	This manuscript
PF166	*MAT****a****/MATα LYS2/lys2Δ CAN1/can1-100 his 3-11,15/his3Δ1* *leu2-3,112/leu2Δ0 MET15/met15Δ0 ura3-1/ura3Δ0 DSK2/Dsk2-GFP* *RAD10/Rad10-YFP UB14/Ub-RFP RAD7/rad7SOCS(L168A, C172A)*	This manuscript
PF167	*MAT****a****/MATα LYS2/lys2Δ CAN1/can1-100 his 3-11,15/his3Δ1* *leu2-3,112/leu2Δ0 MET15/met15Δ0 ura3-1/ura3Δ0 DSK2/Dsk2-GFP* *RAD10/Rad10-YFP UB14/Ub-RFP RAD7/rad7Δ::LEU2*	This manuscript
PF168	*MAT****a*** *his3Δ1 leu2Δ0 met15Δ0 ura3Δ0 Rpn11-TAP rad7SOCS(L168A, C172A)*	This manuscript
W1588-4C	*MAT****a*** *ade2-1 lys2Δ trp1-1 can1-100 his 3-11,15 leu2-3,112 ura3-1*	[48]
W303-1A	*MAT****a*** *ade2-1 lys2Δ trp1-1 can1-100 his 3-11,15 leu2-3,112 ura3-1 rad5-535*	[48]
YFR004W	*MAT****a*** *his3Δ1 leu2Δ0 met15Δ0 ura3Δ0 Rpn11-TAP*	Thermo Scientific TAP-tag library
YHR200W	*MAT****a*** *his3Δ1 leu2Δ0 met15Δ0 ura3Δ0 Rpn10-GFP*	Invitrogen Yeast GFP library
YMR276W	*MAT****a*** *his3Δ1 leu2Δ0 met15Δ0 ura3Δ0 Dsk2-GFP*	Invitrogen Yeast GFP library

a All strains are haploid and derivatives of W303-1A and W303-1B [48] except for TAP-tagged strains which are of the BY4741 background and PF162, PF163, PF164, PF165, PF166 and PF167 which are diploid crosses of the W303-1B and BY4741 strain backgrounds. Additionally, all strains are wild-type for the *RAD5* gene unless otherwise noted.
