# Supplementary material for: Rad7 E3 Ubiquitin Ligase Attenuates Polyubiquitylation of Rpn10 and Dsk2 Following DNA Damage in Saccharomyces cerevisiae
Source: Adv Biol Chem. Author manuscript; Available in PMC 2016 Dec 16. (PMC4832922; doi:10.4236/abc.2015.57021)
Supplement: Supplementary file 1 [file NIHMS749505-supplement-supplement_1.pdf]

Supplemental Figure

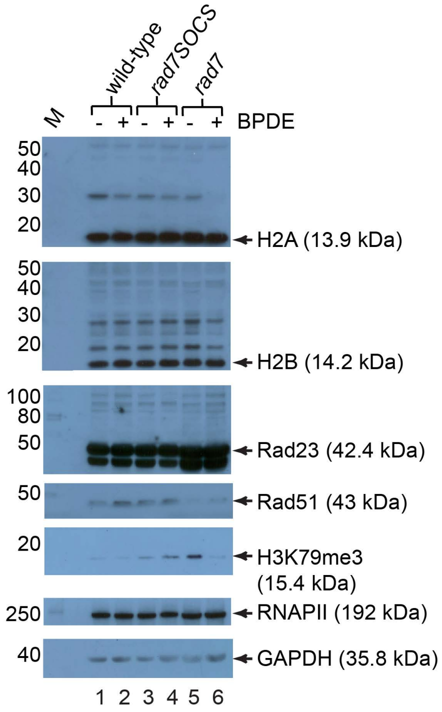

Figure S1. Additional candidates for ubiquitylation that were tested and found not exhibit HMW species that differed between wild-type and *rad7SOCS*. Immunoblots are from the same experiments described for **Figure 3**. Cells from strains PF038-1D, ("wild-type", lanes 1 and 2), PF084-7A, ("*rad7SOCS*", lanes 3 and 4), MGSC104, ("*rad7Δ*", lanes 5 and 6) were cell cycle-arrested at the G2/M boundary, released briefly back into cell cycle, treated with 10 µM BPDE (lanes 2, 4 or 6,"+") for 3 minutes or mock treated (lanes 1, 3 or 5, "-") and disrupted. Normalized protein quantities were analyzed by SDS-PAGE and immunoblotted with the indicated antibodies. "M" indicates MagicMark™ molecular weight marker. The expected native protein size is shown in parentheses next to the antibody name. H2A = Histone 2A, H2B = Histone 2B, Rad23 = Rad23, Rad51 = Rad51, H3K79me3 = Histone 3 K79 trimethylated, RNAPII = RNA Polymerase II. GAPDH is a loading control.
